# Implementation of Home based management of malaria in children reduces the work load for peripheral health facilities in a rural district of Burkina Faso

**DOI:** 10.1186/1475-2875-7-201

**Published:** 2008-10-03

**Authors:** Alfred B Tiono, Youssouf Kaboré, Abdoulaye Traoré, Nathalie Convelbo, Franco Pagnoni, Sodiomon B Sirima

**Affiliations:** 1 Centre National de Recherche et de Formation sur le Paludisme, Ministère de la Santé, Ouagadougou, Burkina Faso, West Africa; 2 Groupe de Recherche et d'Action en Santé (GRAS), Ouagadougou, Burkina Faso, West Africa; 3 UNICEF/UNDP/World Bank/WHO Special Programme for Research and Training in Tropical Diseases, World Health Organization, Geneva, Switzerland

## Abstract

**Background:**

Home Management of Malaria (HMM) is one of the key strategies to reduce the burden of malaria for vulnerable population in endemic countries. It is based on the evidence that well-trained communities health workers can provide prompt and adequate care to patients close to their homes. The strategy has been shown to reduce malaria mortality and severe morbidity and has been adopted by the World Health Organization as a cornerstone of malaria control in Africa. However, the potential fall-out of this community-based strategy on the work burden at the peripheral health facilities level has never been investigated.

**Methods:**

A two-arm interventional study was conducted in a rural health district of Burkina Faso. The HMM strategy has been implemented in seven community clinics catchment's area (intervention arm). For the other seven community clinics in the control arm, no HMM intervention was implemented. In each of the study arms, presumptive treatment was provided for episodes of fevers/malaria (defined operationally as malaria).

The study drug was artemether-lumefantrine, which was sold at a subsidized price by community health workers/Key opinion leaders at the community level and by the pharmacists at the health facility level.

The outcome measured was the proportion of malaria cases among all health facility attendance (all causes diseases) in both arms throughout the high transmission season.

**Results:**

A total of 7,621 children were enrolled in the intervention arm and 7,605 in the control arm. During the study period, the proportions of malaria cases among all health facility attendance (all causes diseases) were 21.0%, (445/2,111, 95% CI [19.3%–22.7%]) and 70.7% (2,595/3,671, 95% CI 68.5%–71.5%), respectively in the intervention and control arms (p << 0.0001). The relative risk ratio for a fever/malaria episode to be treated at the HF level was 30% (0.30 < RR < 0.32).

The number of malaria episodes treated in the intervention arm was much higher than in the control arm (6,661 vs. 2,595), with malaria accounting for 87.4% of all disease episodes recorded in the intervention area and for 34.1% in the control area (P < 0.0001). Of all the malaria cases treated in the intervention arm, only 6.7% were treated at the health facility level.

**Conclusion:**

These findings suggest that implementation of HMM, by reducing the workload in health facilities, might contributes to an overall increase of the performance of the peripheral health facilities.

## Background

Malaria remains the leading cause of child mortality and morbidity in sub-Saharan Africa [1-3]. High malaria transmission intensity, limited access to adequate treatment, increasing parasite resistance to affordable and safe medicines (chloroquine, sulphadoxine-pyrimethamine and amodiaquine); increasing vectors resistance to widely used insecticides, delayed care-seeking and inappropriate treatment at home or at community level, are some of the causes for this deleterious situation.

In 2000, the African Heads of State adopted the access to prompt and effective treatment for at least 60% of those suffering from malaria as one of the goal in reducing the malaria burden for the vulnerable population in Africa [4]. This goal in the context of the limited coverage of the population by health facilities in most of the malaria endemic countries seems quite ambitious and difficult to achieve. Therefore, the Home Management of Malaria (HMM) strategy that has been developed to provide prompt and effective treatment of malaria episodes to those who need it close to their homes represents one of the major strategies that could contribute to achieve this goal [5].

The Roll Back Malaria (RBM) initiative has defined the HMM as an integral part of an overall malaria case management strategy aiming to improve access to treatment for malaria in areas with limited access to health facilities. The strategy aims to improve the ineffective self-medication practices that are very common in malaria-endemic countries; its overall goal is the early recognition and prompt and appropriate response to malarial illness, especially in children under five years of age, in the home or community. It, therefore, empowers communities to respond to malaria illness using effective, good-quality antimalarial medicines through community involvement [5].

Several studies conducted under the leadership of the World Health Organization, have demonstrated its feasibility and efficacy [6-8]. In recent years, with the almost universal development of CQ resistance [9-11], HMM is being promoted with artemisinin-based combination therapy (ACT). Despite some concerns on the widespread use of such drugs at community level [12] recent data have shown that ACT can be used safely within the context of the HMM strategy [13,14].

In Burkina Faso, the HMM strategy has been adopted by the National Malaria Control Programme in an effort to reach the Abuja target. Unfortunately, artemether-lumefantrine (AL)(Coartem^®^), which is to be used at the community level in the context of this strategy, is not yet available in the public sector; the implementation of the strategy is, therefore, facing some delays.

During the high transmission season of malaria, episodes of fever/malaria (from now on operationally defined as 'malaria') in children, account for more than 1/3 of the attendances at first level health facilities. A limited number of health personnel in peripheral health facilities named Centre de Santé et de Promotion Social (CSPS) (usually no more than three staff members including two nurses and one midwife) are assigned to perform a minimal package of activities, including antenatal care services, out-patient management, Expanded Programme of Immunization (EPI), counselling, administrative tasks etc. This results in a very high workload with a potential negative impact on the quality of the services provided.

This study has evaluated the effect of the case management of malaria at a more peripheral level than the health facility – in the community within the context of HMM – on the workload of the health staff operating in first level health facilities.

## Methods

The study was an interventional study, conducted in the rural health district of Saponé located at around 45 km south of Ouagadougou, the capital of Burkina Faso. The climate in the area is characteristic of the Sudanese savannah, with a dry season from November to May and a rainy season from June to October. Malaria transmission is markedly seasonal, with most transmission occurring during the rainy season with an entomological inoculation rate ranging from 50–200 infective bites/person/year. *Plasmodium falciparum *is the most predominant malaria parasite accounting for more than 95% of infections in children less than five years of age. Malaria burden is heaviest among children aged less than 5 years, with every child experiencing an average of two clinical malaria episodes every transmission season.

The health district area covers fourteen community clinics (CSPS), which represent the first level of care in the health system of Burkina Faso. The mean area covered by the catchment area of each CSPS is 9.7 km^2^. Thus, the population has access to the CSPS within an average walking distance of 10 km.

There is no private practice of medicine and drugs selling in the area. Population has legally access to treatment drugs (exclusively generics) including antimalarial drugs through the drug store located in these community clinics; however, there is a parallel market where many other illegally imported drugs are sold.

The study was not randomized. The fourteen community clinics were equally assigned to one of the study arms: seven in the intervention arm and seven in the control arm. Prior to any activity, discussion was conducted with the communities to inform them about the research and seek their assent to participate.

A baseline general census was afterward conducted in both arms to determine the population size, with main focus on the target population for the intervention (children less than five years of age). A health care-seeking behaviour study was conducted to identify, at baseline, whether the two study arms were comparable in regard of this aspect.

Following these baseline activities, in the intervention arm, Community Health Workers (CHWs) and Key Opinion Leaders (KOLs) were identified by their respective communities and trained by the study team to recognize the symptoms of uncomplicated malaria and provide the study treatment, which was artemether-lumefantrine (AL)(Coartem^®^). AL was pre-packaged in two different blister packs by the manufacturer for children aged between 6–35 months (yellow blister pack) and 36–60 months (blue blister pack). CHWs/KOLs were also trained in recognition of symptoms of severity of malaria (prostration, coma, convulsions), as well as other childhood non-malaria diseases, such as pneumonia (cough with fast breathing) or acute diarrhoea, which require immediate referral to the nearest health facility.

In terms of role and responsibilities, while the CHWs and the KOLs were both sharing the responsibility of providing treatment to the sick children, the CHW was playing the additional role of local suppliers of the study drugs to a certain number of KOLs under his/her responsibility. They were responsible for getting the study drugs at the health facility drug store, for their own stock renewal and for supplying the KOLs under their coverage. Thus, CHWs were acting as the link between the local health facility and the KOLs in terms of study drugs supply chain.

The nurses and the drug-store keepers at the health facility have assisted the study team in the training of the CHWs/KOLs. The training has included 189 CHWs/KOLs in the intervention arm. For both arms, 30 nurses (15 in each arm) and 14 drug-stores keepers (7 in each arm) were trained. A post-training survey was conducted to assess CHWs/KOLs knowledge, attitude and practices in order to identify any need of retraining before the study start. An oral interview was conducted with a sample of 40 CHWs and 127 KOLs, of which 56 males (33.5%) and 111 females (66.5%). A questionnaire was used.

The mean age was 33.5 years (range 16 to 60). The majority were farmers (98.8%).

Most of the CHWs/KOLs were able to recognize the malaria symptoms (Table 1). In particular, the main symptom (hot body) was mentioned by the totality of the interviewees.

**Table 1:** Baseline characteristics

	**Intervention arm** **(N = 1021)**	**Control arm** **(N = 1034)**	**P**
**Mean age of the caregivers **(mean ± SD; range)	24.3 ± 6.3 (17; 52)	23.6 ± 5.8 (18; 45)	0.14
**Number of children less than five years **(mean ± SD; range)	1.5 ± 0.01 (1; 3)	1.4 ± 0.01 (1; 3)	0.21
**Knowledge of malaria transmission**			
*Mosquitoes*	282 (27.6%)	291 (28.1%)	0.79
*Others (rain, sun)*	138 (13.5%)	112 (10.8%)	0.06
**Health care-seeking behaviour (first option for treating malaria episodes**)			
*Use of health facility*	289 (28.3%)	278 (26.9%)	0.45
*Self-treatment*	732 (71.7%)	758 (73.3%)	0.45
**Children malaria symptoms mentioned by the caregivers**			
*Hot body*	748 (73.3%)	752 (72.7%)	0.78
*Crying more than usual*	161 (15.8%)	186 (18.0%)	0.18
*Yellowish vomiting*	358 (35.1%)	385 (37.2%)	0.30
*Anorexia*	229 (22.4%)	190 (18.4%)	0.02
*Others*	39 (3.8%)	43 (4.2%)	0.69

The knowledge of the CHWs/KOLs on the requirements for the study drug prescription was also assessed taking the example of the case of a five-months old child; a wrong prescription was noted for 8 CHWs/KOLs (4.2%); the study drug was not indicated for the majority.

Criteria of severity like pallor, fast breathing and convulsions warranting urgent referral were also mentioned by all the workers. At the end of the evaluation session, the need of refresher training on the study drugs prescription was identified. All the CHWs/KOLs benefited from this training.

A study drug (AL) distribution channel was set up. The study drugs were made available by the project to the district main drug store, which supplied each community clinic drug-store. The community clinic drug-store in turn supplied the CHWs acting in their respective villages as main drug-store. The KOLs in each village were supplied by their respective CHWs. For a self-sustainability of the distribution channel, an incentive margin of 10 FCFA (0.024 USD) (per unit of treatment sold) was allowed at each step of the distribution channel.

To initiate the distribution channel, each level was provided proportionally to its need with a seed stock of AL, made available free-of-charge by the project. Final cost of the drug for the caregivers was 100 FCFA (0.24 USD) for the yellow blister pack and 200 FCFA (0.48 USD) for the blue blister pack, whatever place they purchased it (at the CHWs/KOLs level or at the community clinic drug-store level). The standard price of Coartem^® ^was 10 USD in the private sector. AL was not yet available in the public sector at the time of the study.

Despite an effort to bring down the cost of drugs in order to improve as much as possible the financial access for the caregivers, it was anticipated that some vulnerable groups may not be able to afford the cost for the drugs. The CHWs and the KOLs were instructed to deliver the drugs free-of-charge to such groups. The cost was reimbursed to them by the project.

To enhance accessibility of caregivers to CHWs and, therefore, their adherence to the strategy, a ratio of one CHW/KOL per 40 children was adopted. Taking into account the population density of the study area, this ratio offers the advantage that any sick child will be living within less than 25 minutes walk from a CHW/KOLs household.

In the control arm, there were no CHWs/KOLs at the community level. AL was made available in both arms at the health facilities level, at the same cost as in the community [100 Francs CFA (0.24 USD) for the yellow blister pack and 200 Francs CFA (0.48 USD) for the blue blister pack].

The intervention has started in May 2006 and the study drugs were deployed in July 2006. A survey was conducted in October 2006 (at the end of malaria high transmission season) both at community and health facility level to assess activities in terms of malaria cases management of CHWs/KOLs and health centre staff.

### Statistical analysis

Paper forms were used to collect the study data. The completed questionnaires were verified and data double entered electronically and analysed using EPI-INFO (Version 6.04) (CDC, Atlanta, USA and WHO). Standard quality control checks were instituted to ensure the consistency of the data.

The primary outcome to be measured was the number of cases treated for malaria in both study arms. Malaria episodes were defined as the caregiver reporting to the CHWs/KOLs or to the health facility because of his/her child experiencing/having experienced an episode of "*hot body*". No measurement of temperature or of malaria parasitaemia was performed. The use of a case definition based on "hot body" is in line with the WHO HMM strategy and has been previously adopted by Sirima *et al *[4]. From the national health statistics, malaria represent on average 30% of all causes health facility attendance; the minimum sample size required was, therefore, 263 malaria cases treated per study arm over the three months period of the study to detect a reduction of at least 25% between the intervention and the control arm (with 95% confidence interval).

The impact of the intervention in reducing the health facility attendance for malaria episodes in the study population has been calculated using the formula 1 minus the rate ratio (relative risk). Pearson's chi test was used to compare proportions at the significance level of P = 0.05.

### Ethical clearance

The study protocol was reviewed and approved by the ethics committee of the WHO and the National Ethical Committee of Burkina Faso. Appropriate authorities in the district were informed and approval sought from them. Community meetings were held with community leaders in the intervention arm, to discuss with them the purpose of the study and to obtain their adhesion.

## Results

### Background information

**Figure 1:**
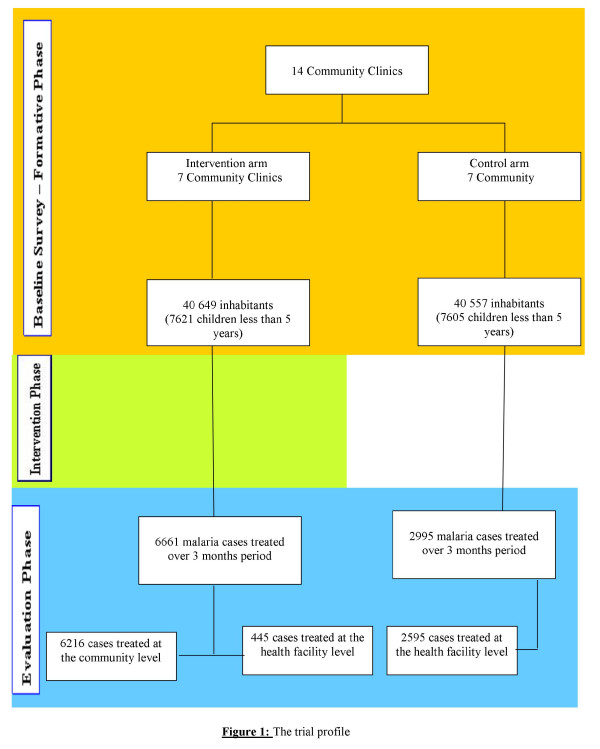
The trial profile.

The baseline census showed a total population of 40,649 in the intervention arm and 40,557 in the control arm. Children aged less than five years were 7,621 and 7,605 respectively. The study profile is provided is figure 1.

To check if there was any difference in health-seeking behaviour in the two arms, a baseline survey was conducted randomly in 1,021 mothers/caregivers in the intervention arm and 1,034 in the control arm. The use of health facilities for the treatment of children fevers was declared as the first option for 28.3% and 26.9%, respectively in the intervention and the control arms (p = 0.45). In terms of clinical signs/symptoms of malaria, the hot body was reported by most of the caregivers (73.3% in intervention arm versus 72.7% in the control arm (p = 0.78). Other signs reported were yellowish vomiting, crying more than usual and anorexia (Table 1).

### Episodes of disease (all causes) treated at health facility (HF) level

From August to October 2006, 2,111 episodes of disease (all causes) in children under the age of five were treated in HF of the intervention arm, and 3,671 in HF of the control arm; this corresponds to an incidence of disease episodes of 27.7% (2,111/7,621) and 48.3% (3,671/7605), respectively (95% CI 26.7%–28.7% and 47.2%–49.4%). Within each of the study arms, there was a trend to an increase of the incidence of disease episodes over the three-month study period (Table 2). The overall workload in the HF of the intervention area was thus reduced by 43%, p << 0.0001.

**Table 2:** Incidence of the diseases episodes at the health facility level

	**Intervention arm **(N = 7621)			**Control arm **(N = 7605)		
		
**Months**	**Nb. of diseases episodes**	**Incidence**		**Nb. of diseases episodes**	**Incidence**	
	*n*	*n/N*	*95% ci*	*n*	*n/N*	*95% ci*
**August**	526	6.9%	(6.3%–7.5%)	1009	13.3%	(12.5%–14.1%)
**September**	575	7.5%	(6.9%–8.1%)	1182	15.5%	(14.7%–16.3%)
**October**	1010	13.3%	(12.5%–14.1%)	1480	19.5%	(18.6%–20.4%)
**Total**	2111	27.7%	(26.7%–28.7%)	3671	48.3%	(47.2%–49.4%)

**Figure 2:**
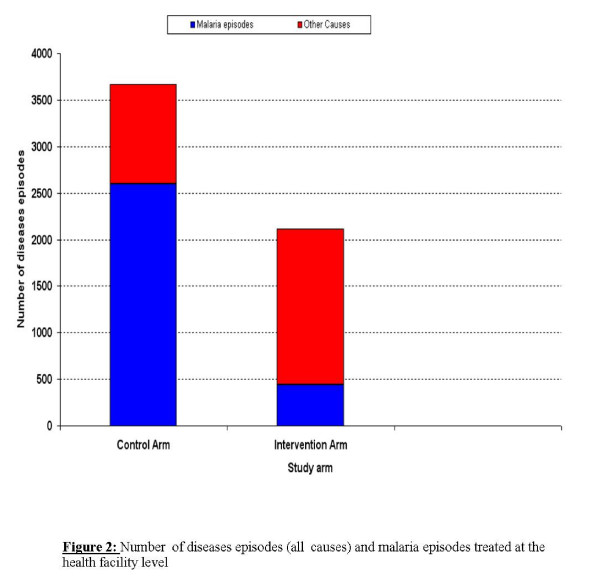
Number of diseases episodes (all causes) and malaria episodes treated at the health facility level.

Non-malaria diseases treated at the health facility level have represented 29.3% in the control arm (1,076/3,671) versus 78.9% (1,666/2,111) in the intervention arm (P << 0.0001). (Figure 2)

### Episodes of malaria treated at HF and community level

A total of 6,661 malaria cases were treated in the intervention arm, where HMM was available, versus 2,595 in the control arm, where treatment was available only at HF level. Thus, the malaria episodes accounted for 87.4% of all disease episodes recorded in the intervention area and for 34.1% in the control area (*P *< 0.0001). Of all the malaria cases treated in the intervention arm, only 445 (6.7%) were treated at the health facility level. Thus, almost 90% of the malaria cases in this arm have been treated by the CHWs/KOLs at the community level.

In general, for the population of children aged less than five years, health facility attendance for malaria episodes has represented 21.0% of overall HF attendance in the intervention arm and 70.7% in the control arm (445/2,111, 95% CI 19.3%–22.7% and 2,595/3,671, 95%CI 68.5%–71.5%, p << 0.0001) (Figure 1). This translates in a relative risk ratio of 30% (0.30 < RR < 0.32) of a malaria episode to be treated at HF level in the intervention arm. The impact of the intervention in reducing the proportion of malaria episodes of the total number of cases managed at the health facility level was, therefore, estimated to be 70.0%.

## Discussion

The outcome measurement has occurred within a relatively short time period after the intervention phase of the study (three months). This period, which corresponds to the end of the high transmission season, when all the players (HF, CHWs/KOLs) have gone through intense activities, is considered as the most accurate to detect significant impact of the intervention on the workload imputable to malaria cases management. A similar time window was used by Sirima et al [8]. Question on whether the findings of this study will be observed if the outcome measurement was done after a longer period is to be considered; however, in the context of the study area, where the malaria transmission is highly seasonal, the HMM strategy will be implemented within the same timeframes used in this study to avoid wastage of resources and minimize the over-treatment of malaria during the low transmission season, when prevalence of the disease is very low. Therefore, the findings of this study could be considered as valid for areas with the same transmission pattern; different assessment timeframe need to be implemented in areas with a longer transmission period, to account for the necessity to sustain the HMM strategy in order to induce an optimum impact on the outcome measured in this study.

The number of malaria episodes treated in the intervention arm was more than double the number of cases treated in the control arm (+156%). This shows that once the option of treating malaria close to home is made available, caregivers make use of it and the number of malaria episodes treated increases. No other reason can explain this finding, as both areas have a similar epidemiological profile and malaria transmission pattern, with comparable health care-seeking behaviour, as shown in the baseline data. The most plausible explanation is thus that the presence of CHWs/KOLs, by increasing the access to an effective treatment in the intervention arm, has boosted the caregiver's willingness to use this new possibility, reducing the use of other sources of treatment.

The presence of the CHWs/KOLs is also the possible explanation to the fact that a significant difference was observed between intervention and control arms in terms of the treatment of non-malaria diseases. Indeed, while a reduction of number of malaria cases treated at the health facility level was observed in the intervention arm, the proportion of non-malaria cases treated there was significantly higher as compared to the control group.

It is possible that, at the community level, the CHWs/KOLs have played the role of "triage staff" for the health facilities, by treating the malaria cases and referring those cases not considered as caused by malaria to the health facility level. This was possible since the training module of the CHWs/KOLs included recognition of the most prevalent non-malaria illnesses in the area (mainly cough with fast breathing proxy for pneumonia, diarrhoeal diseases) and the recommendation of immediate referral to the health system to avoid unnecessary delay in the management of those cases. Also, after two days of home treatment, persistence of the fever warrants a visit to the health facility, since this is more likely to be attributable to other causes than malaria. Unfortunately, no data were collected to document the possible indications for referral.

The feasibility and acceptability of the use of ACTs within the context of HMM has been demonstrated by Ajayi *et al *[13], in a multi-centre study involving four African sites. The present study confirms this finding, as reflected by the high proportion of malaria cases treated by CHWs/KOLs in the intervention arm. The level of knowledge of the CHWs/KOLs of the common clinical symptoms of malaria, and the accuracy in the prescription of study drug was appreciable at the post training survey. There is, therefore, a potential for well-trained CHWs/KOLs to increase the access of the population to appropriate treatment for malaria.

One of the concerns about the wide-scale use of ACTs by the CHWs/KOLs is the possibility of over-prescription of the drug, leading to wastage and possibly increasing the risk of emergence of resistance [12,15,16]. This cannot be excluded in this study. The use of rapid diagnosis tests to target the treatment of true malaria cases with the ACTs is one of the solutions being explored. It has the potential of adding great value to the CHWs/KOLs current performance in managing malaria close to the home, by avoiding the unnecessary delay in the treatment of non-malaria illness, which could be observed with the current presumptive treatment strategy.

The increase in the number of malaria cases treated at the community level is associated with a reduction of the overall workload at health facility level. In the study, the HF attendance rate was lowered by 43% in the intervention arm, with most malaria cases being treated in the community. In the breakdown of disease episodes treated at HF level, malaria accounts for 21% of all attendances in the HF of the intervention arm, while it represents 70.7% in the HF of the control arm. The implications of this finding are obvious.

In developing countries, where malaria burden poses a challenge to the health system, the first level of care for the sick patients is the one that usually bears the high burden of the disease. In this study, the CSPS represents the first point of contact of sick children with the health system, and the workload imposed by the treatment of malaria cases in children by health personnel is not negligible. The intervention has demonstrated the potential of reducing this burden by at least 70.0%.

The health workforce shortage crisis in developing countries is one of the major challenges the health system is facing; it is estimated that to achieve even a modest coverage for essential health interventions, the health worker density required is 2.28 per 1,000 population[17]; in the context of the study area, the actual rates were respectively one nurse per 3,700 population and one doctor per 30,000 [18]. Shortages of health workers in sub-Saharan Africa derive from many causes; among others, one could include past investment shortfalls in pre-service training, morbidity and premature mortality, international migration, premature retirement [19,20].

While various solutions are being explored by policy-makers to circumvent this problem, an intervention reducing by 70% the workload imposed by the treatment of malaria cases in children, could be seen as intermediate solution for malaria endemic countries. Indeed, in the context of limited workforce, the precious time saved could be used by the health staff, for other curative or preventive activities, or to allow a longer nurse-patient contact time, a factor known to be associated with correct management of patients, and higher adherence by caregivers [21].

The home-management of other diseases, such as pneumonia and diarrhoea, is being investigated [22]. There is no doubt that if this could be achieved successfully, as what was observed for malaria, this might contribute to the Millennium Development Goal of reducing children mortality with the increase of the prompt access to appropriate treatment.

## Conclusion

This study has shown that well-trained CHWs/KOLs could provide appropriate treatment for the majority of childhood malaria episodes in their respective communities, and thereby reduce the workload of health staff operating at HF level. This finding provides evidence that a scaling-up of the HMM strategy might contribute to minimize the consequences of workforce shortage at peripheral health facilities level in most of the malaria endemic countries, and possibly improve their performance.

## Competing interests

The authors declare that they have no competing interests.

## Authors' contributions

SBS conceived the study and its design; he coordinated the data collection, the analysis and interpretation of the results and the review of the manuscript. ABT participated to the design of the study, the data collection and analysis and has drafted the manuscript. YK, AT and NC participated in the data collection and data analysis and interpretation. FP contributed in designing the study, monitoring the program implementation and revising the manuscript. All authors have read and approved the final manuscript.
